# Biomonitoring-Based Risk Assessment of Pyrethroid Exposure in the U.S. Population: Application of High-Throughput and Physiologically Based Kinetic Models

**DOI:** 10.3390/toxics13030216

**Published:** 2025-03-16

**Authors:** Nan-Hung Hsieh, Eric S. C. Kwok

**Affiliations:** Human Exposure & Health Effects Modeling Section, Human Health Assessment Branch, Department of Pesticide Regulation, California Environmental Protection Agency, Sacramento, CA 95814, USA; eric.kwok@cdpr.ca.gov

**Keywords:** reverse dosimetry, bayesian, pyrethroids, urinary metabolites, exposure risk

## Abstract

Pyrethroid insecticides have been extensively utilized in agriculture and residential areas in the United States. This study evaluated the exposure risk by age using available biomonitoring data. We analyzed pyrethroid metabolite concentrations in urine using the National Health and Nutrition Examination Survey (NHANES) data. Reverse dosimetry was conducted with a high-throughput model and a physiologically based kinetic (PBK) model integrated with a Bayesian inference framework. We further derived Benchmark Dose (BMD) values and systemic points of departure in rats using Bayesian BMD and PBK models. Margins of exposure (MOE) were calculated to assess neurotoxic risk based on estimated daily oral intake and dose metrics in plasma and brain. Results from both models indicated that young children have higher pyrethroid exposure compared to other age groups. All estimated risk values were within acceptable levels of acute neurotoxic effect. Additionally, MOEs calculated from oral doses were lower than those derived from internal doses, highlighting that traditional external exposure assessments tend to overestimate risk compared to advanced internal dose-based techniques. In conclusion, combining high-throughput and PBK approaches enhances the understanding of human health risks associated with pyrethroid exposures, demonstrating their potential for future applications in exposure tracking and health risk assessment.

## 1. Introduction

Pyrethroids, synthetic insecticides prevalent in residential and agricultural pest control, have raised health concerns due to their extensive use and potential adverse effects [1]. Their usage has significantly increased in recent decades, both in the United States and globally. Growing evidence suggests that exposure to pyrethroids may linked to adverse health effects, including diabetes, neurotoxicity, and cardiovascular disease [2,3,4]. Consequently, researchers have actively monitored and reconstructed pyrethroid exposure among residential and occupational populations [5,6].

Various exposure tools have been developed to estimate both aggregate and cumulative exposure doses of current-use pesticides, including pyrethroids [5,7,8]. In earlier research, Tulve et al. [7] applied three methodologies developed by the United States Environmental Protection Agency (U.S. EPA), including The Standard Operating Procedures for Residential Pesticide Exposure Assessment [9], Draft Protocol for Measuring Children’s Non-Occupational Exposure to Pesticides by all Relevant Pathways [10], and the Stochastic Human Exposure and Dose Simulation (SHEDS) Model for Multimedia, Multipathway Pollutants to estimate cumulative residential exposures to pyrethroids for children aged 4–6 years. Among these approaches, the SHEDS framework was initially developed to estimate children’s residential exposure and dose to chlorpyrifos via dermal contact and non-dietary ingestion [11]. It has been applied to several metals, polychlorinated biphenyls, and pyrethroids for regulatory decisions [7,12,13]. For pyrethroids, Zartarian et al. [5] used permethrin as a benchmark to verify the performance of exposure prediction models, such as the physiologically based kinetic (PBK) model [14] and the SHEDS high-throughput model [15], due to the abundance of experimental exposure and toxicokinetic (TK) data.

Biomonitoring is widely recognized as a reliable method for quantifying human exposure to pyrethroids in populations [16,17,18]. It captures aggregate exposure across all pathways and is an essential tool for chemical risk assessment [19,20]. Health-based guidance values derived from biomonitoring data provide effective and comprehensible evaluations of pyrethroid exposure potential [21]. Commonly used biomarkers for estimating population exposure include urinary metabolites such as 3-phenoxybenzoic acid (3PBA), 4-fluoro-3-phenoxybenzoic acid (FPBA), 3-(2,2-dibromovinyl)-2,2-dimethylcyclopropane carboxylic acid (DBCA), and cis-/trans-3-(2,2-dichlorovinyl)-2,2-dimethylcyclopropane-1-carboxylic acid (DCCA). These biomarkers can either be linked to specific pyrethroid parent compounds or shared among multiple pyrethroids. For example, FPBA and DBCA are specific biomarkers of cyfluthrin and deltamethrin exposure, respectively, while 3PBA is a general metabolite of several pyrethroids that exhibit lower toxicity than the parent compounds.

Biomonitoring data in the U.S. population have been extensively tracked over decades by the National Health and Nutrition Examination Survey (NHANES). A landmark study by Barr et al. [16] analyzed NHANES data collected between 1999 and 2002. The analysis showed that 3PBA was detected in more than 70% of the over 5000 participants, with geometric mean concentrations of 0.29 ng/mL in adults and 0.41 ng/mL in children. Theses data also indicate that children may experience higher pyrethroid exposure. An observational study by Morgan et al. [22] focused on 127 preschool children in homes and daycare centers in six Ohio counties. Results showed 3PBA in 67% of urine samples (median: 0.3 ng/mL; maximum: 33.8 ng/mL). Permethrin was detected most frequently in dust (100%) and hand wipe samples (>78%). Similarly, Trunnelle et al. [23] studied 83 California children aged 2–8 near agricultural areas, reporting a 60% detection rate of 3PBA (median: 0.75 ng/mL), with proximity to fields as a significant exposure factor. However, biomonitoring data alone have limitations in exposure assessment due to factors such as variable exposure frequency, sampling time, and inter-individual variability [24]. Despite these limitations, biomonitoring data can be integrated with computational exposure modeling tools for exposure reconstruction.

TK modeling is an example of a “reverse-dosimetry” approach to estimate the exposure profile of chemicals [25,26] based on the observed chemical levels in human body. U.S. EPA’s ExpoCast project developed a comprehensive high-throughput approach in the Systematic Empirical Evaluation of Models (SEEM) framework to effectively predict the population exposure for chemical prioritization [27,28]. The SEEM framework was used to predict the daily intake based on NHANES biomonitoring data with Bayesian statistical approach. It is noteworthy that the predicted outcomes were compared with other high-throughput approaches, such as SHEDS [15] and the consensus model, which integrates multiple forward exposure model predictions [29].

To better predict chemical intake, PBK models have been applied in several research studies [14,30,31,32,33]. This sophisticated modeling approach considers absorption, distribution, metabolism, and elimination processes in the body. Compared to U.S. EPA’s high-throughput approach, which is based on generic TK model and assumes steady-state equilibrium with mass balance (100% absorption) in a simplified model structure, PBK models provide more accurate parameter estimations and have been validated with experimental TK data. A study conducted by U.S. EPA reported that “The SEEM approach evaluates predictors of exposure based on how well they correlate with estimates of chemical intake rate from biomonitoring” [29]. Thus, the reliability of chemical intake rates inferred from biomonitoring data is crucial for determining model performance. It is essential to examine the quality and representativeness of biomonitoring data alongside model parameters. Given the various predictive tools available, each with strengths and uncertainties in exposure prediction, integrating information from different tools can enhance the scientific rigor of the exposure assessment process [33].

The primary purpose of this study was to understand and track the exposure and dosimetry conditions of pyrethroids in the U.S. population based on available biomonitoring data and assess human health risks. This study employs an open-source and well-developed pyrethroid PBK model to estimate exposure of pyrethroids and their isomers (deltamethrin, cis- and trans-isomers of permethrin, cypermethrin, and cyfluthrin). The U.S. EPA’s high-throughput model outputs were compared with the PBK model predictions, as both reverse dosimetry approaches use the same biomonitoring data (cis- and trans-DCCA, 3PBA, F-PBA, and DBCA) from NHANES. Using multiple biomarkers reduces uncertainties regarding the representation of systemic exposures to the agent [26]. These comparisons offer insights into establishing criteria for accurate exposure prediction in biomonitoring data-driven exposure assessments.

## 2. Materials and Methods

### 2.1. Problem Definition

**Figure 1:**
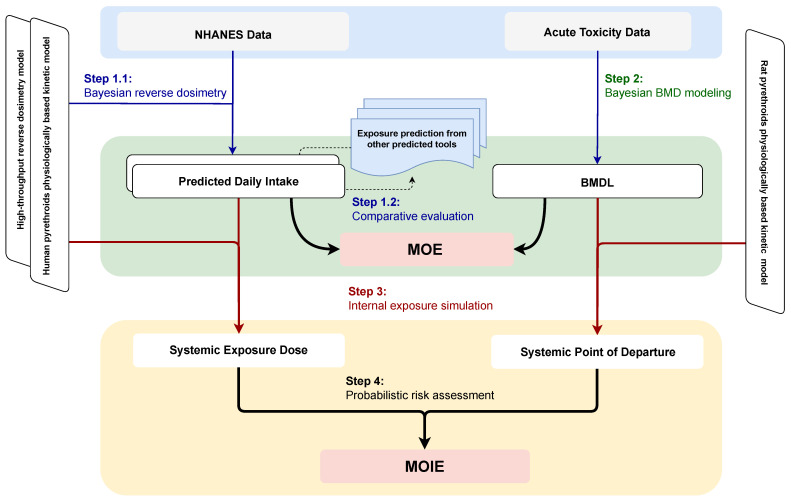
Schematic diagram of the overall study workflow. The study consists of four key steps: (1) using Bayesian reverse dosimetry to estimate daily intake from NHANES biomonitoring data, (2) applying the Bayesian benchmark dose approach to reanalyze dose-response data from animal neurotoxicity studies, (3) simulating systemic exposure dose and determining the point of departure based on external daily intake and benchmark dose thresholds, and (4) probabilistically estimating the margin of internal exposure by integrating predicted exposure levels with effect thresholds. Note: BMD, Benchmark Dose; BMDL, Benchmark Dose Lower Confidence Limit; MOE, Margin of Exposure; MOIE, Margin of Internal Exposure; NHANES, National Health and Nutrition Examination Survey.

Figure 1 illustrates the concept of the study approach. Several uncertainties that may impact the current modeling process and their potential solutions are addressed as follows:

- Model Specification: The uncertainty in model specification was addressed by comparing estimations using two reverse dosimetry modeling approaches. Estimates from other available resources (e.g., official databases, publications) were incorporated for exposure comparison.
- Linkage of Pyrethroids and Metabolites: Five downstream metabolites are associated with four upstream pyrethroid exposures. The pyrethroid PBK model quantified the fraction of metabolites formed from a parent compound by calibrated model parameter using human TK data [34]. The high-throughput approach also addresses the stoichiometric fraction of transformation between multiple parent compounds and their metabolites.
- Non-detect Values below the Limit of Detection (LOD): Nondetect values below the LOD introduce uncertainty in exposure estimation. Bayesian inference with the PBK model was used to predict these non-detect values. The high-throughput method assumes that population urine concentrations are log-normally distributed across all compounds, allowing non-detect values to be imputed using the geometric mean and standard deviation of the simulated distribution.
- Short Half-Lives of Pyrethroids and Metabolites: Pyrethroids and their metabolites typically have short half-lives less than 24 h [35], meaning their concentrations change rapidly over a short period. NHANES spot urine samples were used as surrogates for 24-h average urinary concentrations. This may underestimate the daily dose rate due to random sampling times relative to exposure events [36]. Under the same daily exposure dose but with different dosing time intervals, biomonitoring using spot urine samples and 24-h averages can vary widely. Human urinary half-lives of approximately 12 h have been measured for several pyrethroids following oral exposures [37,38]. Kinetic simulations indicate that once-daily exposures lead to within-day peaks in urinary concentration approximately two- to three-fold higher than the steady-state average concentration for compounds with a half-life of 12 h [39]. Additionally, factors such as hydration status, creatinine excretion rate, and metabolism patterns can impact concentrations by a factor of 2 to 3 [40]. The exposure difference from urine concentration to exposure was addressed by quantifying the variation in observed data and intake prediction.

### 2.2. NHANES Biomonitoring Data

The NHANES database was utilized for exposure reconstruction. Data collection began with the biennial cycle spanning 1999–2001, covering the general U.S. population aged six years and older. Seven subsequent cycles were conducted to investigate pyrethroid exposure. Notably, only the 1999–2000 and 2001–2002 cycles conducted comprehensive surveys of the five pyrethroid metabolites (cis- and trans-DCCA, 3PBA, FPBA, and DBCA). Over time, the metabolites cis-DCCA and DBCA were gradually phased out from the survey, with their last inclusion noted in the 2007–2008 and 2010–2011 cycles, respectively. Detailed information about the metabolites measured by NHANES in each cycle can be founded in Table A1. It is worth noting that the LODs were not consistent across cycles.

### 2.3. The Physiologically Based Kinetic Model

**Figure 2:**
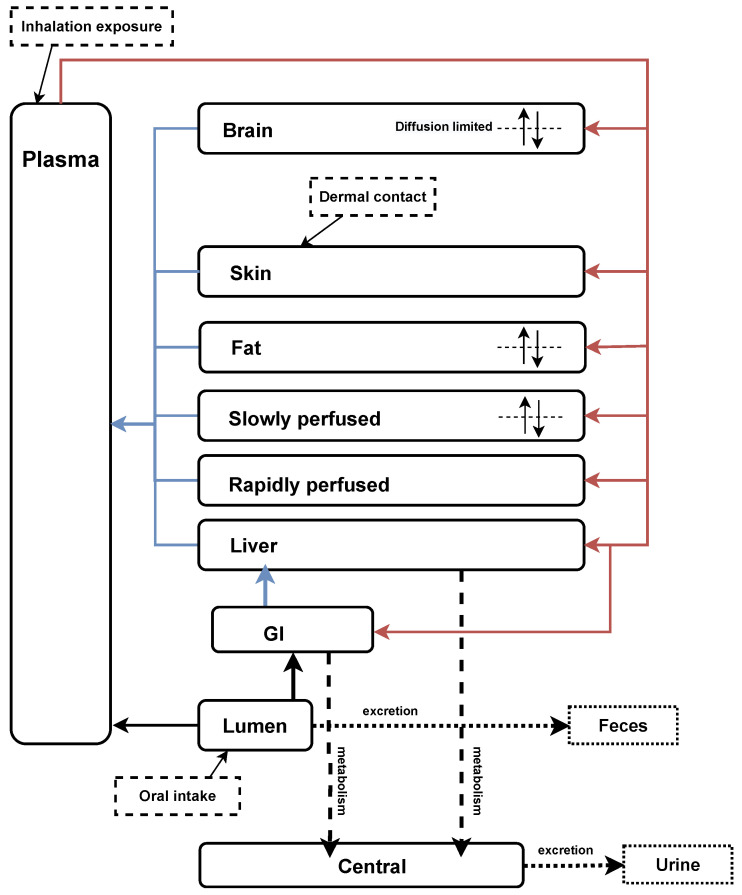
Refined generic PBK model for pyrethroids and their metabolites based on Mallick et al. [42] and Quindroit et al. [34]. The diagram illustrates the PBK model structure, depicting the absorption, distribution, metabolism, and elimination processes of pyrethroids and their metabolites in the human body.

A generic PBK model previously developed by the Council for the Advancement of Pyrethroid Human Risk Assessment [41,42,43] was used to describe the TK of pyrethroids in humans. This model was derived and validated using time-course data from rats [44,45]. We extended the PBK model to verify the human time-concentration profile of metabolites in urine (Figure 2). The structure was based on the generic model for pyrethroids, with the addition of the inhalation exposure route as described by Beaudouin et al. [46] and Quindroit et al. [34]. To validate the refined PBK model, human TK data were adopted from published studies, including exposure routes of oral intake, dermal contact, and inhalation [37,47,48,49]. Throughout the metabolic process, four parent compounds were observed to transform into seven metabolites (Table A2).

As the examined data were spot urine samples without information on the time between exposure and sampling, we assumed all observations were under steady-state exposure conditions. Therefore, each observation reflects the daily average exposure. Consequently, we employed an analytical solution for the PBK model to simplify the estimation of the relationship between intake dose rate and metabolite urine concentration. Under steady-state conditions, only a few parameters are expected to significantly affect metabolite concentration. The description of the critical parameters relevant to urine concentration, along with their corresponding values used in the PBK model can be found in the Table A3. Specifically, physiological parameters such as body weight, urine creatinine, and daily creatinine were found to be associated with urine concentration of pyrethroid metabolites, while factors such as blood flow and organ weight were not considered impactful. Additionally, chemical-specific parameters, including the rate constants of oral absorption and fecal elimination are also directly related to urine concentration of metabolites.

### 2.4. Bayesian Population Modeling

Bayesian population modeling was adopted to predict population exposure to pyrethroids in residential scenarios across all age groups [31,32]. By applying Bayes’ rule, the posterior probability of daily pyrethroid intake was estimated based on the measured urinary metabolite concentrations. The prior distribution of daily intake was calculated, with urinary metabolite concentration treated as likelihood. A uniform (non-informative) distribution of each exposure concentration was assigned in the interval from $10^{-12}$ to $10^{0}$. An inverse gamma distribution was used to describe the prior distribution of population variance [32], with scale and shape parameters equal to 2.25 and 5.0, respectively, assuming both the coefficient of uncertainty and variability in the prior distribution were equal to 2.

For observations above the LOD, the likelihood of the data given the model predictions was assumed to be log-normal, based on the specification of the variance of the residual error. This variance could arise from measurement error, intra-individual heterogeneity, and (or) model misspecification. For observations below the LOD, a normal distribution with a standard deviation equal to LOD/4 was used to describe the deviation between the data and the model, with all observations below the LOD set at 0 and LOD/2, respectively. These settings aim to provide different inferences for nondetected values below the LOD and examine the overall goodness-of-fit. The variances for each of the corresponding residual errors were given half-normal distributions with a standard deviation of 0.3, corresponding to a geometric standard deviation of 1.7.

Physiological parameters, such as body weight, were sourced from NHANES. To reduce computational burden, the chemical-specific parameters (e.g., transformation fractions and isomeric ratios) were set to fixed values based on Quindroit et al. [34]. The intake rate ratio between cypermethrin and permethrin was informed by the California Department of Pesticide Regulation’s (DPR) Pesticide Use Reporting database (https://www.cdpr.ca.gov/docs/pur/purmain.htm, accessed on 14 March 2024) and previous results from the high-throughput model [28].

### 2.5. High-Throughput Model and Other Exposure Prediction Tools

U.S. EPA’s ExpoCast project provides reliable intake estimates based on NHANES biomonitoring data [27]. This approach uses Bayesian statistical methods to infer the probabilistic exposure of intake rates for parent chemicals, assuming a log-normal distribution of product concentrations in NHANES urine samples. The geometric mean and standard deviation were first calculated and used to estimate observations below the LOD. These parameters were verified by comparing the generated cumulative density function (CDF) to the population CDF of the sample data, including quantiles and confidence limits from NHANES data. The statistical characteristics of metabolites were then converted to units of exposure through a reverse dosimetry TK model with a steady-state assumption. For pyrethroids with multiple metabolites, the fractions of each metabolite were also calculated.

In addition to the exposure estimates from biomonitoring data, numerous studies and databases related to pyrethroid exposure and risk assessment were reviewed to compare and summarize the estimated exposure doses. Exposure predictions from the U.S. EPA CompTox Chemicals Dashboard (https://comptox.epa.gov/dashboard/, accessed on 14 March 2024) were adopted to compare with the estimated exposure from the current study. The web-based application includes predictions of daily chemical exposure based on the results of U.S. EPA’s ExpoCast project. The predictions was based on the approaches from Chemical and Products Database, SEEM, and NHANES exposure inferences [50,51].

### 2.6. Exposure Variability

The ratio of the 95th and 50th percentiles (P95/P50) was calculated to assess the variability through the spot urine samples of NHANES biomonitoring data relative to the daily exposure dose. The analysis was conducted for metabolites with over 60% of measurements detected [52]. According to Aylward et al. [36], the variation of observed exposure dose is similar to the predicted dose and can be used to assess the misestimation of intake in reverse dosimetry. Based on this concept, we estimated the underlying daily dose distributions to inform the interpretation of population biomonitoring data in the context of uncertainty quantification. The P95/P50 ratios of predicted intake doses were also calculated and compared with the ratio of biomonitoring measures. The resulting ratio provides an indication of the degree of over- or under-prediction of variation in doses as reflected by the variation in biomarker concentrations.

### 2.7. Bayesian Benchmark Dose Modeling

This study focuses on acute exposure and risk assessment due to (1) the spot urine samples from NHANES not being suitable for interpreting repeated long-term exposure, (2) the short half-lives of pyrethroids reflecting recent rather than continuous exposure, and (3) the lack of robust sub-chronic and chronic toxicity data for exposure risk assessment.

Acute oral toxicity data were sourced from open literature [53], as well as from Benchmark dose (BMD) data analysis conducted by U.S. EPA [54]. The data were selected because of rigorous data standard required for federal regulatory purpose. Neurotoxicity was assessed by locomotor activity. The Bayesian BMD was applied to reanalyze the toxicity data using a web-based system [55].

The default models for continuous data and non-informative priors were used to fit the dose-response data, using Markov chain Monte Carlo (MCMC) simulation. Three Markov chains were calculated with 30,000 iterations per chain. The model distributions were estimated using the last half of the iterations (15,000 iterations per chain). The determination of BMD was based on model averages from 8 continuous models and using the posterior model weights. One standard deviation of the response from the control was chosen for BMD as suggested by U.S. EPA.

### 2.8. Risk Estimates

The margin of exposure (MOE) and margin of internal exposure (MOIE) approaches were used to calculate human health risk, based on the (1) PBK-estimated daily oral intake and (2) the corresponding internal exposure dose metrics. The MOE is a quantitative tool used by DPR and most regulatory agencies to determine the potential risk arising from exposure to a pesticide or other chemicals [46,56]. It is defined as the ratio of the critical point of departure (POD) value, derived from the critical acute, sub-chronic, or chronic studies to the estimated human exposure. The resulting value is compared to the acceptable or target MOE, with values at or above the target MOE are generally considered protective against the toxicity of the compound in question. For this analysis, the MOE was calculated by dividing the Benchmark dose lower confidence limit (BMDL) by the exposure estimate of pyrethroid.

Compared to the MOE, the MOIE is a relatively advanced exposure assessment strategy that relies on the sophisticated PBK model to provide a robust evaluation for decision-making [57]. Because of the correlation between brain concentration and acute neurotoxic effects [58], this study calculated the MOIE by using PBK-derived systemic BMDL divided by dose metrics, such as peak concentration (Cmax) and area under the curve (AUC) in the plasma and the brain. The rat PBK model from Song et al. [41] was applied with the BMDL to simulate the time-concentration curve based on a single oral intake in Wolansky et al. [53], and to estimate the dose metrics. The refined life-stage human PBK model was applied with Monte Carlo simulations, to estimate the distribution of the four dose metrics according to the estimated daily exposure dose.

The PBK model builds on the comprehensive sensitivity analysis reported by Mallick et al. [42]. The study identified the brain partition coefficient and the unbound fraction in plasma as the most sensitive parameters affecting pyrethroid distribution, followed by metabolic clearance and permeability. Key physiological parameters, such as body weight, cardiac output, hematocrit, hepatic plasma flow, and fat volume, were also found to be highly influential. In this research, compound-specific values and uncertainty levels were applied to these previously validated parameters as outlined in Table A4.

### 2.9. Computational Settings and Evaluation

The PBK model, along with its input conditions, was developed for use in the GNU MCSim software (version 6.1.0) [59]. All other statistical analyses and visualizations were conducted using the latest version of the R programming language (version 4.4.0) with the relevant packages. Additionally, the U.S.EPA-developed R package bayesmarker [28] was used to estimate the exposure intake dose rate based on the high-throughput approach. For both Bayesian modeling approaches, three independent Markov Chains were run to estimate the posterior distributions. Convergence was assessed as recommended by Gelman and Rubin [60]. The goodness of fit was evaluated by assessing the posterior distribution of measurement error.

## 3. Results

### 3.1. Exploratory Analysis of the Biomonitoring Data

The current analysis encompassed a total of 18,665 individuals across seven biennial cycles, from 1999–2000 to 2015–2016. The 2015–2016 cycle introduced an additional survey focusing on children under six years old, but covering only three metabolites. To facilitate a comparison of exposure doses with prior research, the population was segmented into five distinct age groups: <6, 6–11, 12–19, 20–65, and >65 years. The grouping was the same as the U.S. EPA’s ExpoCast project.

Initial analysis of summary statistics revealed notable patterns in detection rates of various biomonitoring results (Table A5). The consistently high detection rate of 3PBA was particularly striking because the detection rate exceeded 60% across all observed years. Conversely, the detection of DBCA remained consistently low with registering at less than 2%. A significant observation was the trend in FPBA detection, which exhibited a discernible increase over the last three biennial cycles (2010–2016), indicating a noteworthy shift in exposure levels over time. Under the same LOD conditions, the detection rate of 3PBA showed an increasing trend from around 70% before 2010 to 95% in the latest survey (2015–2016). During the 2007–2016 period, the detection rates of FPBA and trans-DCCA increased from 6.5% to 13.2% and from 15.8% to 37.5%, respectively.

**Figure A1:**
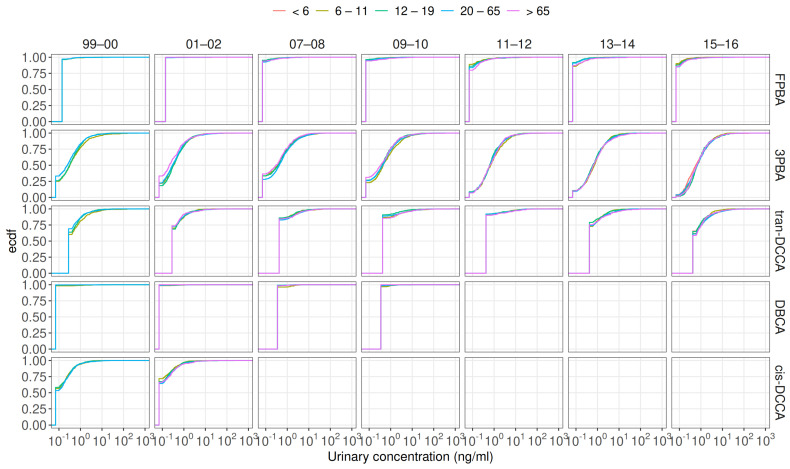
Cumulative distribution of urinary pyrethroid metabolite concentrations across different age groups.

While examining the cumulative distribution of urine concentrations across different age groups (Figure A1), no significant disparities were evident. However, a clear upward trend in detection rates emerged over the same three biennial cycles for 3PBA, suggesting a potential temporal influence on exposure levels.

### 3.2. Model Evaluation

**Figure 3:**
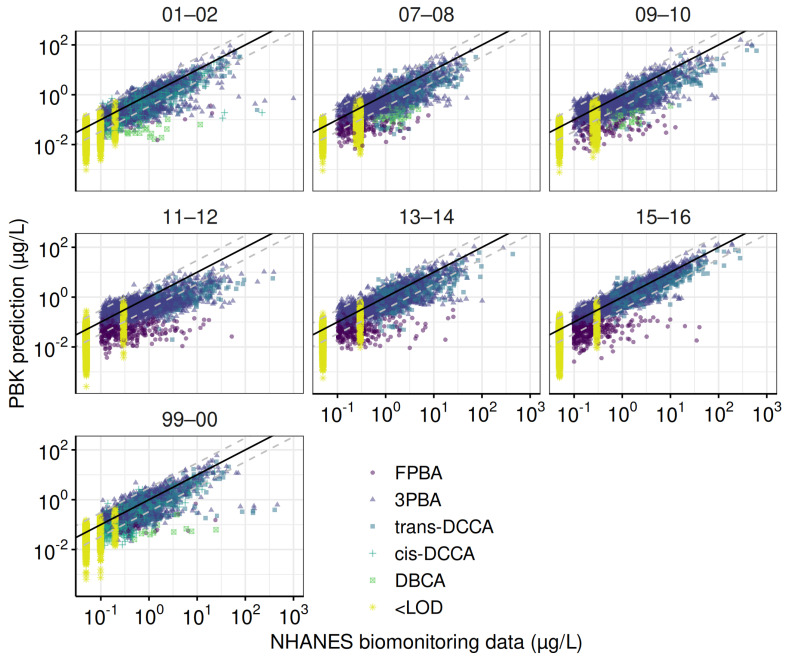
Goodness–of–fit evaluation for the total population across NHANES cycles. These plots compare observed and predicted urinary concentrations of pyrethroid metabolites for the total population across NHANES cycles. Predictions are based on parameters from the final Markov Chain Monte Carlo iterations. The solid line represents a perfect match between observed and predicted values, while dashed lines indicate error margins within a ten-fold difference.

**Figure A2:**
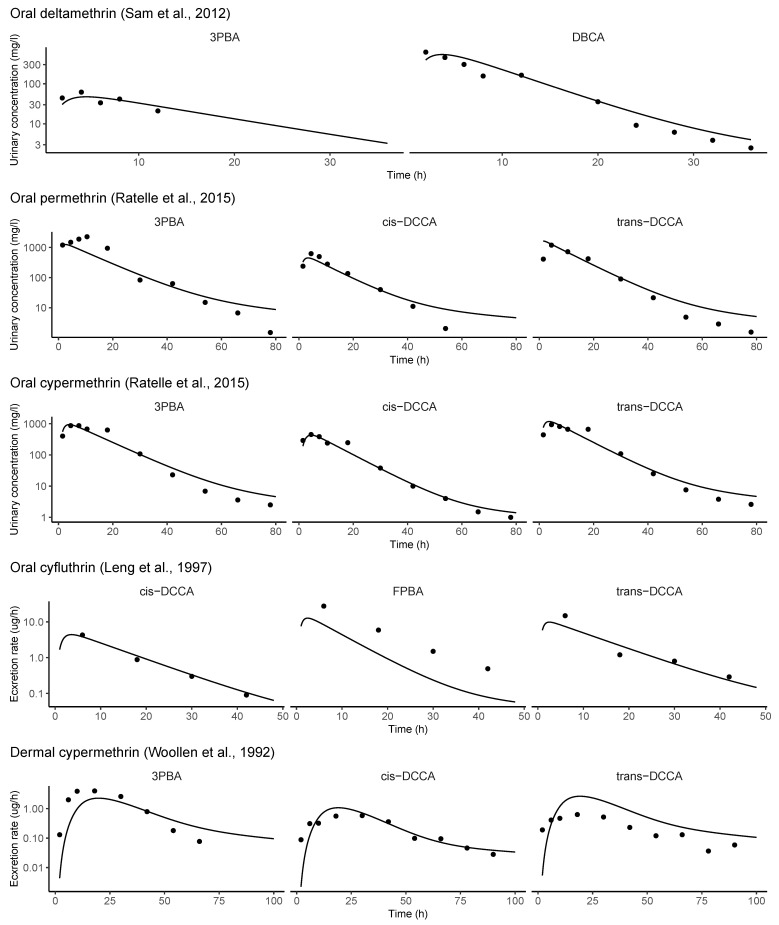
Evaluation of model performance of refined PBK model with human toxicokinetic experiments [37,38,48,49,74].

The refined PBK model performed well with human TK experiments, which included over a hundred data points from 14 TK profiles of 5 pyrethroid metabolites (Figure A2). Using the default parameter values, 99% of the predictions fell within a 10-fold range, approximately 75% within a 3-fold range, and 65% within a 2-fold range of the observed values. The calibrated PBK model was used to estimate the daily intakes of the four pyrethroid parent compounds. The observed biomonitoring data were compared with the corresponding model predictions (Figure 3). Overall, the refined PBK model performed better during the earlier biennial cycles (1999–2000 and 2001–2002), where all five metabolites were fully investigated.

**Figure 4:**
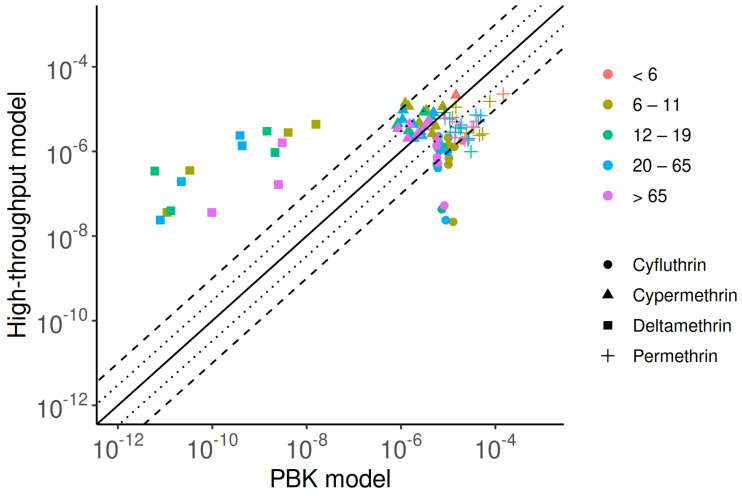
Comparison of exposure predictions from the PBK model and high-throughput approaches, highlighting similarities and discrepancies. The solid line represents a perfect match between the two methods, while dotted and dashed lines indicate errors within three- and ten-fold differences, respectively.

Figure 4 shows a comparison of model predictions from the PBK and high-throughput models. The estimated median geometric mean intake rates reveal that permethrin and cypermethrin demonstrate strong consistency between the two approaches, with over 80% of estimates differing by less than 10-fold. For cyfluthrin, 60% of predictions fall within this range. In contrast, predictions for deltamethrin show significant disparity in intake rate estimates. The PBK model generally produces lower estimates, typically 2 to 5 orders of magnitude below those of the high-throughput model. For example, in children (6–11 years old), the median predicted intakes of deltamethrin were $3.65\times 10^{-7}$ mg/kg-d from the high-throughput model and $3.31\times 10^{-11}$ mg/kg-d from the PBK model. This discrepancy arises largely from limitations in the PBK model’s capacity to estimate deltamethrin intake rates across the final three biennial periods (2010–2016), primarily due to the unavailability of DBCA data. Combined with limited investigation and a low detection rate of DBCA, these factors lead to inaccurate exposure predictions for deltamethrin and reduce consistency in the results. In contrast, cyfluthrin exhibits significantly higher intake estimates from the PBK model. For the 2001–2002 cohort, the predicted intakes for children were $1.27\times 10^{-5}$ mg/kg-d by the high-throughput model and $2.73\times 10^{-8}$ mg/kg-d by the PBK model.

The cross-comparison of exposure dose predictions based on different tools is detailed in Supplementary Materials File S1. Briefly, using average food residue data (mg/kg food) from the United States Department of Agriculture (USDA) Pesticide Data Program (PDP) database (2002–2011) and consumption data (g food/kg bw/day) for individuals aged 6–79 from the U.S. EPA Food Commodity Intake Database (2005–2010), Aylward et al. [39] estimated the exposures of permethrin, cypermethrin, and deltamethrin to be $1.83\times 10^{-3}$, $6.1\times 10^{-4}$, and $1.9\times 10^{-6}$ mg/kg-d, respectively. In the U.S. EPA report, intake estimations are based on the Dietary Exposure Evaluation Model (DEEM) Software with Food Commodity Intake Database, which uses 2003–2008 food consumption data from the USDA’s National Health and Nutrition Examination Survey, What We Eat in America (NHANES/WWEIA). The SEEM approach, validated by NHANES data, provides exposure estimates similar to those based on biomonitoring data. According to the latest U.S. EPA reports, across all age groups and pyrethroid pesticides, the population-adjusted dose (PAD) shows that cyfluthrin and beta-cyfluthrin presents the highest acute dietary risk estimate for children aged 1–2, which used 96% of the acute PAD. The risk estimates of PAD are reported at the 99.9th and 50th percentiles for acute and chronic exposure. By contrast, biomonitoring data-driven intake rates have %PAD values of less than 2%. Additionally, using food consumption data, the predicted intake of pyrethroids is about an order of magnitude higher than predictions from biomonitoring data. Regardless of the tools employed, children typically exhibit the highest estimated exposure levels compared to other age groups. It is important to note that DPR’s own process of assessing risk from dietary exposure does not use 50th percentile approach for estimating chronic exposure but rather more refined consumption estimates based on the available data.

### 3.3. Exposure Variability, Trend, and Similarity

**Figure 5:**
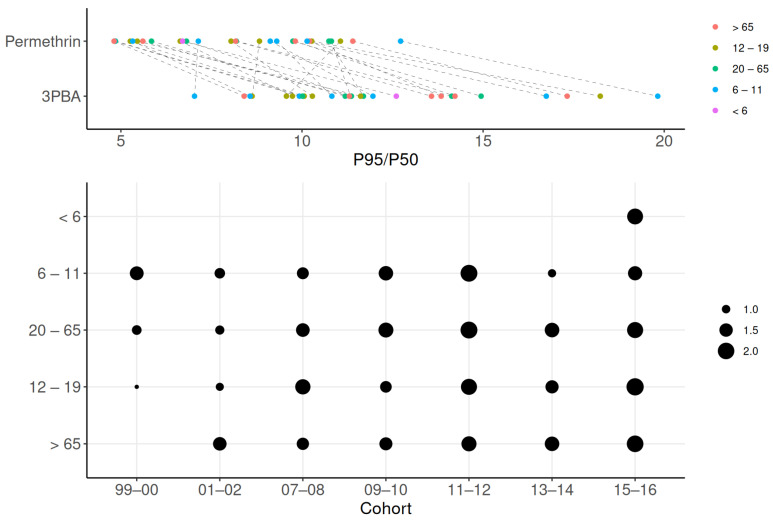
Variability in exposure estimates across age groups. Variability in exposure is shown using P95/P50 ratios for 3PBA and permethrin across age groups. Greater variability is observed in the high differences between permethrin and 3PBA in the top panel, as well as in the larger dots depicted in the lower panel.

We selected 3PBA to assess variability and differences in reverse dosimetry because it was the only compound with a detection rate over 60%. Preliminary examination showed that the coefficient of variation for 3PBA was over 33%, which correspond to the expected concerns about data reliability [52]. Figure 5 shows the differences in P95/P50 across age groups for 3PBA and permethrin. The highest estimated ratio (19.8) for 3PBA was observed in the 6–11 age group during 1999–2000. In comparison, the ratio for permethrin in the same group was estimated to be 12.6, indicating a 1.6 times difference between the P95/P50 values, possibly due to other unknown factors (e.g., inter-individual variability). Across all cohorts and age groups, the P95/P50 for 3PBA ranged from 7.0 (6–11 years old; 2013–2014) to 19.8, reflecting wide inter-individual variation in dose rates and the low half-life and exposure interval ratio. Overall, the ratio for 3PBA is higher than that for its parent compound, permethrin, for which P95/P50 ranged 4.8 (6–11 years old; 2011–2012) to 11.4 (>65 years old; 2001–2002). The factor of P95/P50 difference between 3PBA and permethrin ranged from 0.86 (12–19 years; 1999–2000) to 2.09 (12–19 years old; 2015–2016), with no significant differences across age groups.

**Figure 6:**
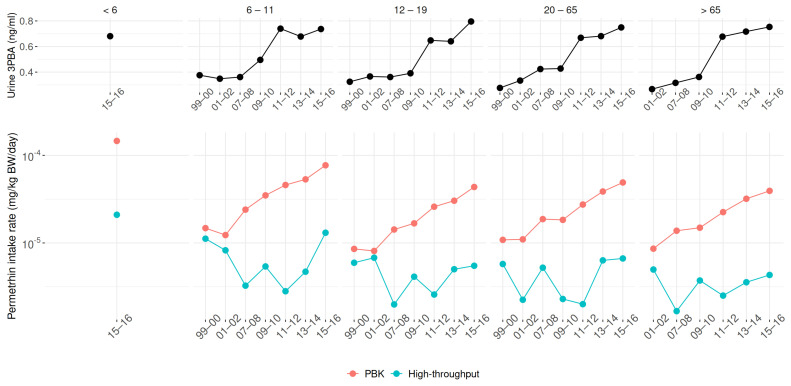
Trends in urinary 3PBA (**top**) and derived permethrin daily intake (**bottom**) from 1999 to 2016. Predicted intakes are shown separately for PBK and high-throughput models.

Our study also investigated the exposure trend based on the 3PBA biomonitoring data and its related parent compound. As mentioned, the detection rate has significantly increased in recent years. Figure 6 shows the geometric mean of 3PBA and the predicted daily intake rate of permethrin, estimated by the PBK and high-throughput models across all cohorts from 1999 to 2016. Permethrin was selected due to the highest detection rate of 3PBA and the completeness of the investigation across all cohorts. According to the urine 3PBA data, there was an increasing exposure trend from 1999 to 2016. Compared to earlier surveys, the geometric mean of urine 3PBA was about two-fold higher in the 2015–2016 investigation than in the 1999–2000 study. In our prediction, the PBK model showedan exposure trend with an intake rate increment of less than an order of magnitude. However, unlike the PBK model, the high-throughput approach could not provide an exposure trend. Nonetheless, a common finding was that children under 6 years old had the highest exposure compared to other population subgroups. This result could not be obtained by exploring only the urine 3PBA data.

**Figure 7:**
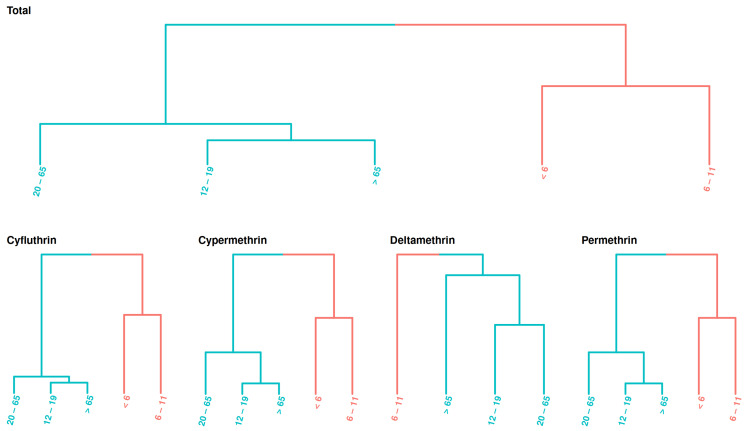
Exposure similarity across age groups. Age groups are arranged by class, highlighting similar exposure levels across NHANES cycles.

To identify specific exposure patterns, the median exposure estimates for each population group from each cycle were used to examine exposure similarity (Figure 7). The results show that the children aged <6 and those between 6–11 years exhibited similar exposure patterns, compared to the other four age groups over 12 years old. Overall, our results indicate higher exposure levels in younger populations, consistent with other prediction tools shown in Supplementary Materials File S1.

### 3.4. Risk Assessment

Exposure predictions from the PBK model revealed that the highest annual exposure to deltamethrin occurred in the 6–11 age group during the 2009–2010 NHANES cycle. For permethrin and cyfluthrin, the highest exposure levels were observed in children under 6 years old during the 2015–2016 cycle. To establish a conservative protective strategy, exposure values from the top 1% of each group were used to calculate the MOE and the systemic exposure-derived MOIE. Bayesian inference estimates showed that the geometric mean population oral intake rates for deltamethrin, permethrin, and cyfluthrin were $3.83\times 10^{-5}$ mg/kg-day (GSD = 1.09), $2.25\times 10^{-3}$ mg/kg-day (GSD = 1.13), and $6.79\times 10^{-5}$ mg/kg-day (GSD = 1.11), respectively. The intake rate for cypermethrin was estimated at $2.25\times 10^{-4}$ mg/kg-day based on the intake rate of permethrin.

Using the Bayesian BMD model, the estimated BMDL were determined as 1.79 mg/kg for deltamethrin, 39.17 mg/kg for permethrin, 5.48 mg/kg for cypermethrin, and 1.22 mg/kg for cyfluthrin. Rat PBK modeling provided Cmax and AUC values in plasma and brain tissue for each compound. For deltamethrin, the plasma Cmax and AUC were 118.2 ng/mL and 1174.1 ng-h/mL, respectively, while the brain values were 34 ng/g and 475 ng-h/g.

Following Wolansky’s findings of a 40% cis and 60% trans isomer composition for permethrin, the plasma Cmax and AUC for cis-permethrin were 1605.5 ng/mL and 15,822.6 ng-h/mL, with brain values of 459.8 ng/g and 6400 ng-h/g. For trans-permethrin, the plasma Cmax and AUC were 503 ng/mL and 4926.1 ng-h/mL, while brain values were 143.8 ng/g and 1996.4 ng-h/g.

In the absence of specific TK data and PBK parameters for cyfluthrin and cypermethrin, chemical-specific parameters from deltamethrin were used to estimate dose metrics. For cis-cyfluthrin, the plasma Cmax and AUC were 32.18 ng/mL and 319.8 ng-h/mL, while brain values were 9.26 ng/g and 129.4 ng-h/g. For trans-cyfluthrin, the plasma Cmax and AUC were 48.3 ng/mL and 479.8 ng-h/mL, with brain values of 13.9 ng/g and 194.1 ng-h/g.

For cis-cypermethrin, the plasma Cmax and AUC were 152.2 ng/mL and 1510.8 ng-h/mL, while brain values were 43.7 ng/g and 611.2 ng-h/g. For trans-cypermethrin, the plasma Cmax and AUC were 210.4 ng/mL and 2087.9 ng-h/mL, with brain values of 60.5 ng/g and 845.7 ng-h/g.

**Figure 8:**
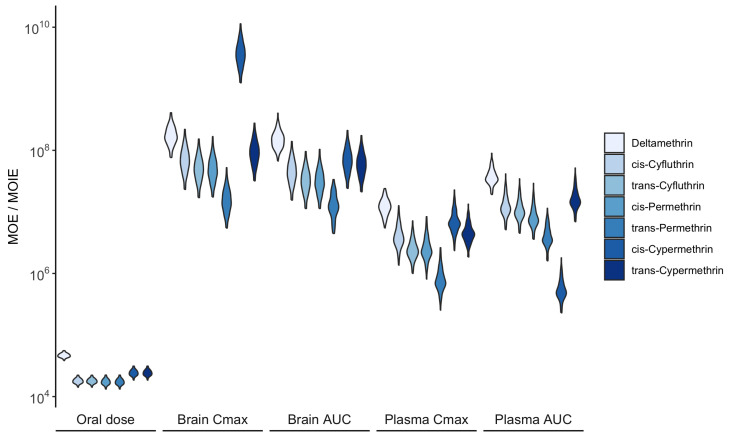
Distribution of the MOE and MOIE for acute neurotoxic effects calculated from daily oral and corresponding systemic exposure, respectively. Higher values indicate a lower exposure risk.

Figure 8 illustrates the estimated probability distributions of the MOE and MOIE for pyrethroid pesticides, based on external exposure and the associated dose metrics. Overall, all estimated values exceeded $1.0\times 10^{4}$, indicating an acceptable level of acute risk under exposure conditions derived from biomonitoring data. Notably, MOEs calculated from oral doses were lower than MOIEs derived from internal doses, highlighting that conventional external exposure assessments tend to overestimate risk compared to advanced internal dose-based techniques.

## 4. Discussion

In this study, a PBK model was successfully refined to reconstruct exposure patterns from publicly available biomonitoring data on pyrethroid metabolites within the U.S. population. This methodology aligns with the previous approaches used for methylmercury and chloroform, where the focus was on a single model without comparative exposure assessments, potentially compromising reliability [31,32]. This study rectifies this by benchmarking PBK model predictions against other exposure prediction tools, thus reinforcing the PBK model’s robustness and applicability in exposure assessment.

It is noteworthy that human TK data were reused from earlier model development, which is critical for robust predictions in model verification. While major structural changes where not introduced to the existing model, it performance was validated with the same TK data to ensure model reliability. The refined PBK model demonstrated similar predictability to the global pyrethroid model by Quindroit et al. [17], with approximately 65% and 75% of predictions falling within 2-fold and 3-fold error intervals, respectively. The model developed by Quindroit et al. [17] applied only to simulate the pyrethroids concentration for adult group. Our refined model aims to apply the exposure prediction to different age groups, including children.

This study primarily compares daily intake estimates with the high-throughput modeling approach utilizing NHANES biomonitoring data for exposure estimation. Bayesian inference was applied to quantify uncertainty in both methods. Previous studies excluded outliers to avoid acute exposure under atypical conditions [3], whereas this study included all observations, providing a comprehensive depiction of exposure. The Bayesian probabilistic approach’s flexibility was instrumental in achieving this goal. The Bayesian method also has the capability to include the data that is below the LOD in order to provide proper inference.

Generally, the PBK model tends to estimate higher exposure levels than the high-throughput approach due to the inclusion of chemical-specific bioavailability parameters. Lower detection rates for key metabolites, such as DBCA and FPBA, led to inconsistencies in predictions for deltamethrin and cyfluthrin compared to permethrin and cypermethrin, with less than 60% of predictions within a 10-fold difference. The lack of DBCA data in the more recent cycles (after 2009–2010) further complicated these predictions. Despite these challenges, both methods consistently indicated higher pyrethroid exposure levels in children compared to other age groups, corroborating findings from other studies using dietary intake from food commodities in exposure prediction. Newly available NHANES data (2015–2016) indicate that children aged 3–5 have the highest exposure compared to the older age groups.

Advanced research by Stanfield et al. [61] supports the high-throughput approach’s capability to detect exposure trends. In addition, using food consumption data, the predicted intake of pyrethroids is approximately ten times higher than the predictions derived from biomonitoring data. This discrepancy suggests that biomonitoring assessments, which rely on detecting major urine metabolites, may underestimate exposure. This underestimation occurs if these metabolites, assumed to be stable first-generation products, without undergoing further metabolism. For instance, exposure to cyfluthrin might be underestimated if its metabolite, DCCA, continues to be metabolized further.

Our exposure prediction closely aligned with the findings of the France study [17], which also employed a PBK approach. The study estimated median daily intakes of $3.4\times 10^{-5}$, $8.1\times 10^{-4}$, $2.0\times 10^{-5}$, and $1.8\times 10^{-5}$ mg/kg-day for deltamethrin, permethrin, cyfluthrin, and cypermethrin, respectively. Additionally, no risks were anticipated from the reconstructed pyrethroid exposures. Furthermore, a study in China found that metabolite levels among 481 infants were similar to or slightly higher than those in the U.S. population [62].

Previous research shows that chronic NOAELs are similar to or higher than the acute POD used in this study [63]. For deltamethrin, the chronic dog oral intake study (1 mg/kg/day) and Wolansky et al. [53] (0.99 mg/kg) have very similar PODs, which are among the lowest available. Therefore, a POD of approximately 1 mg/kg should be protective of both acute and long-term effects. It is crucial to note that the BMDLs used in the current study are higher than the lower confidence limit of threshold dose estimated by [53], where benchmark response was a 30% reduction in motor activity versus one standard deviation of the control value selected by U.S.EPA. However, the Bayesian statistical approach has been proven to have higher capability in determining regulation than the traditional frequentist method for estimating robust BMD in risk analysis [64].

This study specifically focused on acute exposure risks. Due to the short half-life of pyrethroids, predicting exposure based on a steady-state assumption introduces significant uncertainty. Previous research has indicated that children’s exposures are more likely to be episodic rather than resulting in a steady-state condition [65]. As noted earlier, risk estimates derived from reverse dosimetry-based exposure and associated endpoints are generally sufficient to address risks from repeated exposures. However, they are less suitable for reconstructing source-specific exposure patterns over short and long durations. Specifically, based on the risk assessment conducted by U.S. EPA [63,66,67,68,69], there appears to be no increased toxicity from repeated or chronic exposure to pyrethroids. Therefore, the risk estimates derived from acute studies are adopted to protect against risks from repeated exposures. Compared to scenario-based approaches, such as the one conducted by U.S. EPA [68], the reverse dosimetry approach employed in this work may not adequately assess health risks associated with pathway-specific exposures over the short and long durations. While U.S. EPA’s cumulative risk assessment identified no risks of concern for pyrethroid pesticides, the study was conducted over a decade ago. The POD values used were based on benchmark dose modeling of data from Functional Observational Battery study, which are substantially higher (up to an order of magnitude) than the BMD modeled values presented in studies based on reduced motor activity by Wolansky et al. [53,70]. Given advancements in risk assessment methodologies and the availability of new toxicological data, there is a need for an updated cumulative risk assessment to ensure that current exposure scenarios and potential risks are adequately characterized.

In a risk assessment based on data from the Canadian Health Measures Survey and using neurotoxic effects as the endpoint, Faure et al. [52] reported that the hazard quotient of 3PBA exceeded 1. While 3PBA is a metabolite of several pyrethroids with varying toxicities, the metabolite itself is considered less toxic. As a result, reverse dosimetry is required to investigate the parent compounds present in target tissues. This research showed that in a similar geographical context, all estimated MOEs for the U.S. population exceeded $1.0\times 10^{4}$, contrasting with the findings of the Canadian study and indicating that pyrethroid exposure among the general U.S. population is at an acceptable level.

Unlike most studies that conduct health risk assessments using urinary concentrations or intake dose rates, this study applied a PBK model to assess risk based on dose metrics in the brain and plasma, which are directly associated with neurotoxic health effects. This study approach is similar to a study by Arnot et al. [71], who applied the internal threshold of toxicological concern to compare the no observed effect level from oral intake and whole-body exposure.

There are certain limitations in the PBK dose reconstruction. In the refined PBK model, the parameters were based on earlier studies without further modification. Consequently, because the original model was constructed primarily based on animal and human studies of deltamethrin and permethrin, parameters for cyfluthrin and cypermethrin were sourced from in vitro and in silico studies or extrapolated from the other two pyrethroids [34], resulting in observational and predictive differences over a factor of 3. Exposure predictions from biomarkers are generally lower than those from dietary sources. Due to the relatively short half-lives of pyrethroids, it is likely to underestimate intake rates through spot urine samples from NHANES. Additionally, Barr et al. [16] indicated that morning samples had significantly higher concentrations than those collected in the afternoon and evening.

Sampling time, frequency, and detection limits significantly influence the accuracy and interpretation of biomonitoring data, affecting predictions of chemical intake [35]. Specifically, (1) the timing of sample collection impacts measured concentrations of chemicals and their metabolites. For pyrethroids, which have short biological half-lives, urinary levels fluctuate markedly depending on exposure timing. Sampling too early or too late may misrepresent actual exposure. (2) The frequency of sampling determines how well exposure trends are captured. Spot urine samples reflect only recent exposures and may not represent chronic levels, whereas 24-h collections average fluctuations for a more reliable picture. Hays et al. [35] noted that peak urinary concentrations can exceed steady-state levels by twofold, making single-spot samples less suitable for comparison to reference values. (3) Analytical sensitivity governs the detection of low-level exposures. High detection limits may miss subtle but significant exposures, underestimating risk, while overly sensitive limits might detect trace, biologically irrelevant levels, potentially raising undue concern. Together, these factors shape the reliability of biomonitoring results and their use in exposure prediction.

The PBK model provides an opportunity to estimate chemical-specific human variability TK adjustment factors to replace the default uncertainty factor of 3.16 [72]. However, this method did not work in the present study as the only uncertainty/variability is from exposure. In the modeling process, unlike high-throughput model, the PBK model requires adequate animal experiment data to develop a robust model in exposure prediction.

Regarding the data availability, the newly accessible biomonitoring data from NHANES used in the current study lags by nearly a decade. This delay in data availability limits its ability to reflect recent exposure trends in a timely manner. Consequently, alternative data sources, such as pesticide use reports (e.g., PUR data from DPR) and regional investigations, can serve as valuable tools for understanding and predicting exposure trends.

Based on results, the current study highlights three key findings: (1) Both PBK and high-throughput models consistently identify children as the population with the highest pyrethroid exposure, aligning with prior studies that support this finding (e.g., Barr et al. [16]). (2) The exposure trend reconstructed by the PBK model closely aligns with biomonitoring data, as shown in Figure 6. In contrast, the high-throughput model struggles to capture exposure trends, likely due to its simplified structure and the omission of chemical- and physiological-specific parameters. Stanfield et al. [28], key developers of the high-throughput approach, noted that missing data for over half of the metabolites increases uncertainty and complicates exposure prediction. (3) Compared to the high-throughput approach, the PBK model offers more details insights by integrating systemic exposure data, providing more robust information for exposure assessment.

This study refined the pyrethroid PBK model and identified potential future applications. For example, it is preferable to use a PBK model to derive relative potency factors based on internal dosimetry. Quindroit et al. [17] and Thépaut et al. [73] applied the PBK model in cumulative risk assessment, though their studies were based on intake dose rate rather than internal dose in target organs related to toxicity endpoints. The updated model was used to estimate and update the Tier 1 provisional biomonitoring equivalent in Aylward et al. [39] for assessing population urinary 3PBA data. The Tier 1 value was used for evaluating population biomonitoring data for 3PBA in the context of a conservative cumulative risk assessment for a group of pyrethroid compounds.

The primary objective of this study is to leverage advanced computational approaches, integrated with biomonitoring data, to evaluate human health risks associated with pyrethroid exposure. This methodology can enhance the clarity and accuracy of exposure assessments. While the findings suggest that current pyrethrois exposure levels fall within acceptable thresholds based on existing risk assessment frameworks, a detailed exploration of regulatory implications, such as reassessing exposure limits or refining risk assessment methodologies, extends beyond the scope of this research. Such considerations would require additional analyses, which were not the focus of this investigation.

In conclusion, the refined PBK model substantially enhances the understanding of pyrethroid-associated health risks, providing reliable estimates and identifying critical exposure trends. This study underscores the model’s potential for broader applications in health risk assessments and highlights the need for ongoing research and regulatory updates to safeguard public health. By continuously improving exposure models and integrating comprehensive biomonitoring data, vulnerable populations can be better protected from potential health risks associated with pyrethroid insecticides.

## Data Availability

All biomonitoring data from NHANES can be found in the Centers for Disease Control and Prevention’s website (https://www.cdc.gov/nchs/nhanes, accessed on 3 March 2023). The prediction from high-throughput reverse dosimetry model can be found in the U.S.EPA GitHub repository (https://github.com/USEPA/CompTox-HumanExposure-bayesmarker, accessed on 1 January 2023). The Bayesian BMD analysis and results are available for deltamethrin (https://benchmarkdose.com/run/d29c89e5-1ac9-4408-8788-806cf924f615/, accessed on 10 March 2025), β-Cyfluthrin (https://benchmarkdose.com/run/de55bf8c-0c2e-4b78-9040-cc1930e2506d/, accessed on 10 March 2025), cypermethrin (https://benchmarkdose.com/run/95f15b77-b36f-4714-80bc-846ad2127049/, accessed on 10 March 2025), and permethrin (https://benchmarkdose.com/run/2b98df53-151c-403e-8301-07972703eaa9/, accessed on 10 March 2025). All data and code for modeling and analysis in this study is publicly available at (https://github.com/nanhung/toxics-13-00216, accessed on 10 March 2025).

## Supplementary Materials

The following supporting information can be downloaded at: https://www.mdpi.com/article/10.3390/toxics13030216/s1, Table S1: Summary of exposure predictions from this study and other predicted approaches.

## Abbreviations

The following abbreviations are used in this manuscript:

3PBA	3-Phenoxybenzoic Acid
BMD	Benchmark Dose
BMDL	Benchmark Dose Lower Confidence Limit
DBCA	3-(2,2-Dibromovinyl)-2,2-Dimethylcyclopropane Carboxylic Acid
DCCA	3-(2,2-Dichlorovinyl)-2,2-Dimethylcyclopropane-1-Carboxylic Acid
DPR	Department of Pesticide Regulation
EPA	Environmental Protection Agency
FPBA	4-Fluoro-3-Phenoxybenzoic Acid
LOD	Limit of Detection
MOE	Margin of Exposure
MOIE	Margin of Internal Exposure
NHANES	National Health and Nutrition Examination Survey
NOAEL	No-Observed-Adverse-Effect Level
PAD	Population-Adjusted Dose
PBK	Physiologically Based Kinetic
POD	Point of Departure
RfD	Reference Dose
SHEDS	Stochastic Human Exposure and Dose Simulation
TD	Toxicodynamics
TK	Toxicokinetics
USDA	United States Department of Agriculture

## Appendix A

### Appendix A.1. Tables

**Table A1:** Measured pyrethroids metabolites and the limits of detection (μg/L) across each NHANES cycle.

	1999–2000	2001–2002	2007–2008	2009–2010	2011–2012	2013–2014	2015–2016
3PBA	0.1	0.1	0.1	0.1	0.1	0.1	0.1
FPBA	0.2	0.2	0.1	0.1	0.1	0.1	0.1
trans-DCCA	0.4	0.4	0.6	0.6	0.6	0.6	0.6
DBCA	0.1	0.1	0.5	0.5			
cis-DCCA	0.1	0.1					

**Table A2:** Pyrethroid parent compounds and corresponding metabolites.

	3PBA	FPBA	DBCA	cis-DCCA	Trans-DCCA
Cyfluthrin		x		x	x
Cypermetrhin	x			x	x
Deltamethrin	x		x		
Permethrin	x			x	x

**Table A3:** Summary of key PBK parameters related to urine pyrethroid metabolites.

Parameter	Description	Unit	Value	Reference
BW	Body Weight	kg	Observed	
UrineCreatinine	Urine Creatinine	mg creatinine/dL urine	Observed	
DailyCreatinine	Daily Creatinine	mg creatinine/day	Estimated	[28]
DLM_KA	Uptake rate of deltamethrin	/h	1.51	[34]
cisPRM_KA	Uptake rate of cis-permetrhin	/h	0.52	[34]
tranPRM_KA	Uptake rate of trans-permethrin	/h	1.3	[34]
cisCPM_KA	Uptake rate of cis-cypermethrin	/h	0.52	[34]
tranCPM_KA	Uptake rate of trans-cypermethrin	/h	1.3	[34]
cisCYF_KA	Uptake rate of cis-cyfluthrin	/h	0.52	[34]
tranCYF_KA	Uptake rate of trans-cyfluthrin	/h	1.3	[34]
DLM_Kfec	Fecal excretion rate of deltamethrin	/h	0.59	[34]
cisPRM_Kfec	Fecal excretion rate of cis-permetrhin	/h	0.39	[34]
tranPRM_Kfec	Fecal excretion rate of trans-permethrin	/h	0.85	[34]
cisCPM_Kfec	Fecal excretion rate of cis-cypermethrin	/h	0.39	[34]
tranCPM_Kfec	Fecal excretion rate of trans-cypermethrin	/h	0.85	[34]
cisCYF_Kfec	Fecal excretion rate of cis-cyfluthrin	/h	0.39	[34]
tranCYF_Kfec	Fecal excretion rate of trans-cyfluthrin	/h	0.85	[34]
DLM_3PBA	Metabolic fraction of deltamethrin to 3PBA	-	0.15	[34]
DLM_DBCA	Metabolic fraction of deltamethrin to DCCA	-	0.73	[34]
cisPRM_3PBA	Metabolic fraction of cis-permethrin to 3PBA	-	0.37	[34]
cisPRM_DCCA	Metabolic fraction of cis-permethrin to DCCA	-	0.37	[34]
transPRM_3PBA	Metabolic fraction of trans-permethrin to 3PBA	-	0.85	[34]
transPRM_DCCA	Metabolic fraction of trans-permethrin to DCCA	-	0.61	[34]
cisCPM_3PBA	Metabolic fraction of cis-cypermethrin to 3PBA	-	0.16	[34]
cisCPM_DCCA	Metabolic fraction of cis-cypermethrin to DCCA	-	0.32	[34]
transCPM_3PBA	Metabolic fraction of trans-cypermethrin to 3PBA	-	0.39	[34]
transCPM_DCCA	Metabolic fraction of trans-cypermethrin to DCCA	-	0.57	[34]
cisCYF_FPBA	Metabolic fraction of cis-cyfluthrin to FPBA	-	0.1	[34]
cisCYF_DCCA	Metabolic fraction of cis-cyfluthrin to DCCA	-	0.27	[34]
transCYF_FPBA	Metabolic fraction of trans-cyfluthrin to 3PBA	-	0.23	[34]
transCYF_DCCA	Metabolic fraction of trans-cyfluthrin to DCCA	-	0.35	[34]

**Table A4:** Distribution of PBK parameters used in Monte Carlo simulation for exposure risk assessment.

Parameter Type	No. of Parameter	SD	Truncation (±nxSD)
Plasma unbound fraction	1	0.1	2
Partition coefficient	7	0.3	3
Permeability area cross product	3	0.3	3
Uptake and fecal excretion rate	2	0.4	3
Clearance rate	2	0.4	3

**Table A5:** Summary of detection rate (%) for each prethroid metabolites across each NHANES cycle.

	1999–2000	2001–2002	2007–2008	2009–2010	2011–2012	2013–2014	2015–2016
3PBA	68.74	77.04	65.02	72.34	91.00	88.25	95.70
FPBA	3.04	0.49	6.55	4.63	15.34	10.18	13.02
trans-DCCA	33.24	28.31	15.83	11.62	7.65	24.62	37.49
DBCA	0.63	0.88	1.46	1.48			
cis-DCCA	41.59	33.08					
